# Contamination of *Clostridium perfringens* in soy sauce, and quantitative microbial risk assessment for *C. perfringens* through soy sauce consumption

**DOI:** 10.1002/fsn3.2182

**Published:** 2021-02-19

**Authors:** Yeongeun Seo, Yewon Lee, Sejeong Kim, Jimyeong Ha, Yukyung Choi, Hyemin Oh, Yujin Kim, Min Suk Rhee, Yohan Yoon

**Affiliations:** ^1^ Department of Food and Nutrition Sookmyung Women's University Seoul Korea; ^2^ Risk Analysis Research Center Sookmyung Women's University Seoul Korea; ^3^ Department of Biotechnology College of Life Sciences and Biotechnology Korea University Seoul Korea

**Keywords:** @risk, *Clostridium perfringens*, dose‐response, predictive model, quantitative microbial risk assessment, soy sauce

## Abstract

The objective of this study was to conduct QMRA (quantitative microbial risk assessment) of *Clostridium perfringens* through soy sauce consumption. Four hundred and ninety soy sauce samples from markets were analyzed to detect *C. perfringens*. Temperature and time were also measured during transportation and display of soy sauce. A primary model was developed by fitting the Weibull model to the *C. perfringens* cell counts in soy sauce at 7–35°C, and δ (the time needed to decrease 1 log CFU/ml) and *ρ* (curve shape) were calculated. The parameters were analyzed, using the Exponential model (secondary model) as a function of temperature. The consumption amount and percentage of soy sauce were surveyed, and a dose‐response model was searched. Using all collected data, a simulation model was prepared in the @RISK program to estimate the probability of *C. perfringens* foodborne illness by soy sauce consumption. *C. perfringens* were negative in 490 samples. Thus, the initial contamination level was estimated to be −2.9 log CFU/ml. The developed predictive models showed that *C. perfringens* cell counts decreased during transportation and display. The average consumption amounts, and the percentage of soy sauce were 7.81 ml and 81.2%, respectively. The simulation showed that the probability of *C. perfringens* foodborne illness by consumption of soy sauce was 1.7 × 10^–16^ per person per day. Therefore, the risk of *C. perfringens* by consumption of soy sauce is low in Korea.

## Graphical abstract

This study aimed to soy sauce consumed in Korea and conduct QMRA. It shows that the risk of foodborne *C. perfringens* infection by consumption of soy sauce can be considered low in Korea.

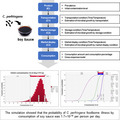

## 1 INTRODUCTION

Soy sauce is one of the seasonings fermented with soy and is consumed in many countries. The annual production of soy sauce in Korea is more than 251,172 t (Ministry of Food & Drug Safety, 2018), and the 13,699 t is exported to the United States, Russia, and China (Korea Agro‐Fisheries & Food Trade Corporation, 2018). However, the possibility of *Clostridium perfringens* has been continuously raised. *C. perfringens* is one of the most common causes of foodborne disease outbreak in the United States and Korea, alongside norovirus, *Salmonella*, *Campylobacter*, and *Escherichia coli* (Centers for Disease Control & Prevention, 2017; Ministry of Food & Drug Safety, 2020).

*Clostridium perfringens* are anaerobic bacteria and exist as vegetative cells or spores in food. Cooking (60°C) kill the vegetative cells, but the spores. These spores can germinate into vegetative cells at 20–30°C, which is the temperature for soy sauce storage, and the vegetative cells produce toxin in the intestinal tract (McClane et al., 2012). Hence, the contamination of *C. perfringens* in soy sauce and the risk of *C. perfringens* by soy sauce consumption should be evaluated.

Quantitative microbial risk assessment (QMRA) is a scientific evaluation method for estimating risks caused by microorganisms, including hazard identification, exposure assessment, hazard characterization, and risk characterization. Thus, to estimate the risk of *C. perfringens* contamination in food, QMRA is appropriate (Codex Alimentarius Commission, 2015; Jeong et al., 2018; Lee et al., 2019; WHO, 2002). Also, risk assessment of *C. perfringens* should be assessed through risk assessment based on predictive models. Therefore, the objectives of this study were to investigate the contamination of *C. perfringens* in soy sauce and to evaluate the risk of the pathogen through soy sauce consumption.

## 2 MATERIALS AND METHODS

### 2.1 Investigation of *C. perfringens* contamination in soy sauce

Four hundred and ninety soy sauce samples were purchased from supermarkets in Korea. One milliliter of the soy sauce was spread‐plated on tryptose sulfite cycloserine agar (TSC; Difco) plates and incubated at 37°C for 18–24 hr under the anaerobic condition created by an anaerobic pack (Oxoid; Basingstoke) in anaerobic jar. The resulting colonies were analyzed, using a compact VITEK^®^2 ANC ID card (BioMérieux). When the result indicated acceptable or higher, it was determined as positive for *C. perfringens*. The prevalence data of *C. perfringens* were fitted to the Beta distribution (α_1_, the number of positive samples + 1; α_2,_ tested total samples‐positive samples + 1), and the initial contamination level (CFU/ml) was then evaluated, using the equation: [–LN(1–Beta(α_1_, α_2_))/ml] (Sanaa et al., 2004; Vose, 1998).

### 2.2 Development of predictive models

*Clostridium perfringens* strains KCCM (Korean Culture Center of Microorganisms) 12098, KCCM40946, KCCM40947, and KCTC (Korean Collection for Type Cultures) 5101 were cultured in 10 ml cooked meat medium (CMM; Oxoid, Basingstoke, Hampshire, UK) at 37°C for 24 hr under the anaerobic conditions. Three‐milliliter aliquots of the cultures were inoculated in 30 ml Duncan and strong medium (15.0 g proteose peptone, 10.0 g sodium phosphate, 4.0 g raffinose, 4.0 g yeast extract, 1.0 g sodium thioglycolate, and 50.0 ml 0.51 mM caffeine in 1 L distilled water) and anaerobically incubated at 37°C for 48 hr. The *C. perfringens* strains were harvested by centrifugation at 1,912 × *g* and 4°C for 15 min. The supernatant was discarded, and the cells were washed twice with phosphate buffered saline (PBS; pH 7.4, 8.0 g NaCl, 1.5 g NaHPO_4_, 0.2 g KH_2_PO_4_, and 0.2 g KCl in 1 L distilled water). The cell pellets were resuspended in 3 ml PBS, and the strains were mixed to be used as the inoculum. The *C. perfringens* inoculum was inoculated to 10 ml soy sauce in a conical tube to achieve a final concentration of 3.0 log CFU/ml, and stored at 7, 15, 25, and 35°C for up to 5 days. During storage, 10 ml of the soy sauce was diluted with 20 ml buffered peptone water (BPW; Dickinson and Company), and it was vortexed. The homogenates were spread‐plated on TSC agar, and the plates were incubated anaerobically at 37°C for 24 hr. Typical black colonies with halos were counted, and the *C. perfringens* cell count data were fitted to the Weibull model. The equation was as follows;

(1) $$\log\left(N\right)=\log\left(N_{0}\right)‐{\left(\frac{\mathrm{time}}{\delta }\right)}^{\rho }.$$

where N is cell counts, N_0_ is initial cell counts, δ is treatment time for the first decimal reduction and *ρ* is curve shape (Mafart et al., 2002). To describe the effect of temperature on δ, a secondary model was developed with the equation as follows;

(2) $$\delta =a\times \exp\left(k\times T\right).$$

Where *α* is constant, *k* is the rate constant and *T* is storage temperature. To evaluate the model performance, *C. perfringens* was inoculated in soy sauce as described above, and the samples were stored at 20 and 30°C. During storage, *C. perfringens* cells were counted as described above to obtain the observed values. The differences between the observed data and predicted data derived from the developed predictive models for 20 and 30°C were quantified by calculating the root mean square error (*RMSE*) values, bias factor (*B_f_*), and accuracy factor *(A_f_)* as follows (Ha et al., 2019);

(3) $$RMSE=\sqrt{1/n\times \sum {\left(\text{observed data}‐\text{predicted data}\right)}^{2}}.$$

(4) $$B_{f}=10^{\sum log\;\left(\text{predicted data}/\text{observed data}\right)/n}.$$

(5) $$A_{f}=10^{\left(\sum \left|\begin{array}{c} log\;\left(\text{predicted data}/\text{observed data}\right) \end{array}\right|/n\right)}.$$

where *n* represents the number of data points. The *RMSE* is the square root of the variance of the residuals. It indicates the absolute fit of the model to the data–how close the observed data points are to the predicted values. The *B_f_* shows by how much models over‐predict (*B_f_* > 1) or underpredict (*B_f_* < 1), and *A_f_* shows by how much the predicted data differ from the observed data (Yoon et al., 2011).

### 2.3 Investigations for soy sauce storage conditions and consumption amounts

The temperature of the transport vehicle was collected by measuring the internal temperature of the transport vehicle of four companies in a retail market. Storage temperature and time data for soy sauce at retails were collected through interviews with managers at retail markets. The transport temperature data were analyzed by @RISK 7.6 (Palisade Corp.) to determine an appropriate probabilistic distribution. To have the daily consumption amounts and percentage for the soy sauce. The data from the Korea National Health and Nutrition Examination Survey 2017 were used. The consumption data were analyzed, using the @RISK 7.6 to determine an appropriate probabilistic distribution. The consumption percentage of soy sauce was calculated by dividing the number of total respondents (6,628 people) in the survey to the number of respondents (5,382 people) who consumed soy sauce.

### 2.4 Search for dose response model

To evaluate the response of a human to *C. perfringens* dose, when people intake soy sauce contaminated with *C. perfringens* consumption, dose response models were searched through published articles.

### 2.5 Preparation of simulation model

A simulation model was then prepared in Excel^®^ (Microsoft Corp.) spreadsheet, with the initial concentration of *C. perfringens*, predictive models, probabilistic distributions for the transportation time and temperature used for the predictive models to describe the bacterial growth, a probabilistic distribution of consumption amount, consumption percentage, and the dose‐response model. The simulation model eventually calculated the probability of *C. perfringens* foodborne illness by soy sauce consumption per person per day with Monte Carlo simulation in @RISK.

## 3 RESULTS AND DISCUSSION

### 3.1 Prevalence and initial contamination levels of *C. perfringens*

**FIGURE 1:**
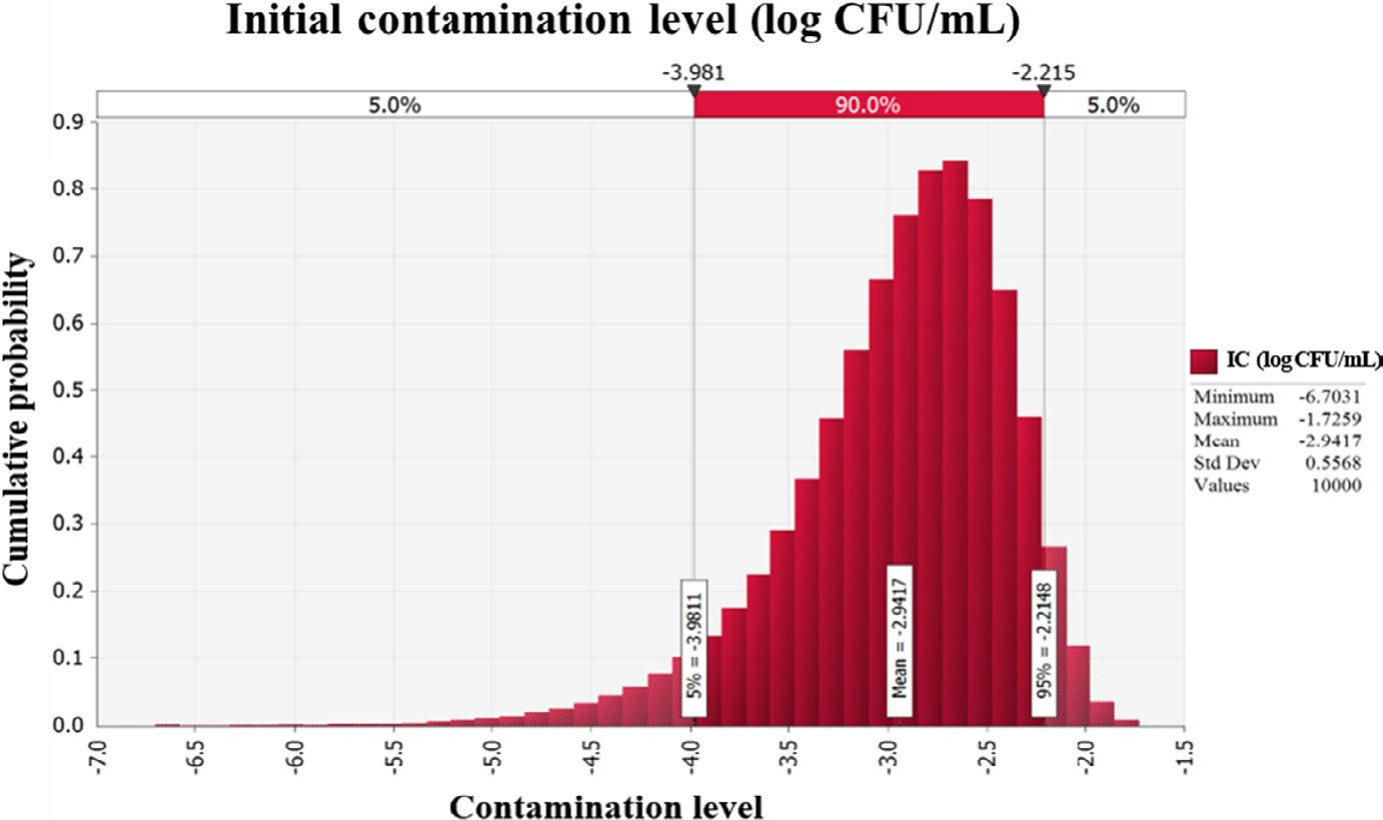
Cumulative probability of simulated initial contamination level of *Clostridium perfringens* in soy sauce

Of 490 samples, *C. perfringens* cell counts were below the detection limit (1 log CFU/ml) in all samples. Because there were no *C. perfringens* positive samples, contamination levels need to be estimated. Thus, the Beta distribution was used to estimate the initial contamination level of *C. perfringens* in soy sauce (Vose, 1998), and the Beta distribution (RiskBeta [1,491]) was used to, and initial *C. perfringens* contamination levels were estimated to be −2.9 log CFU/ml (Figure 1).

### 3.2 Predictive model to describe the kinetic behavior of *C. perfringens*

**FIGURE 2:**
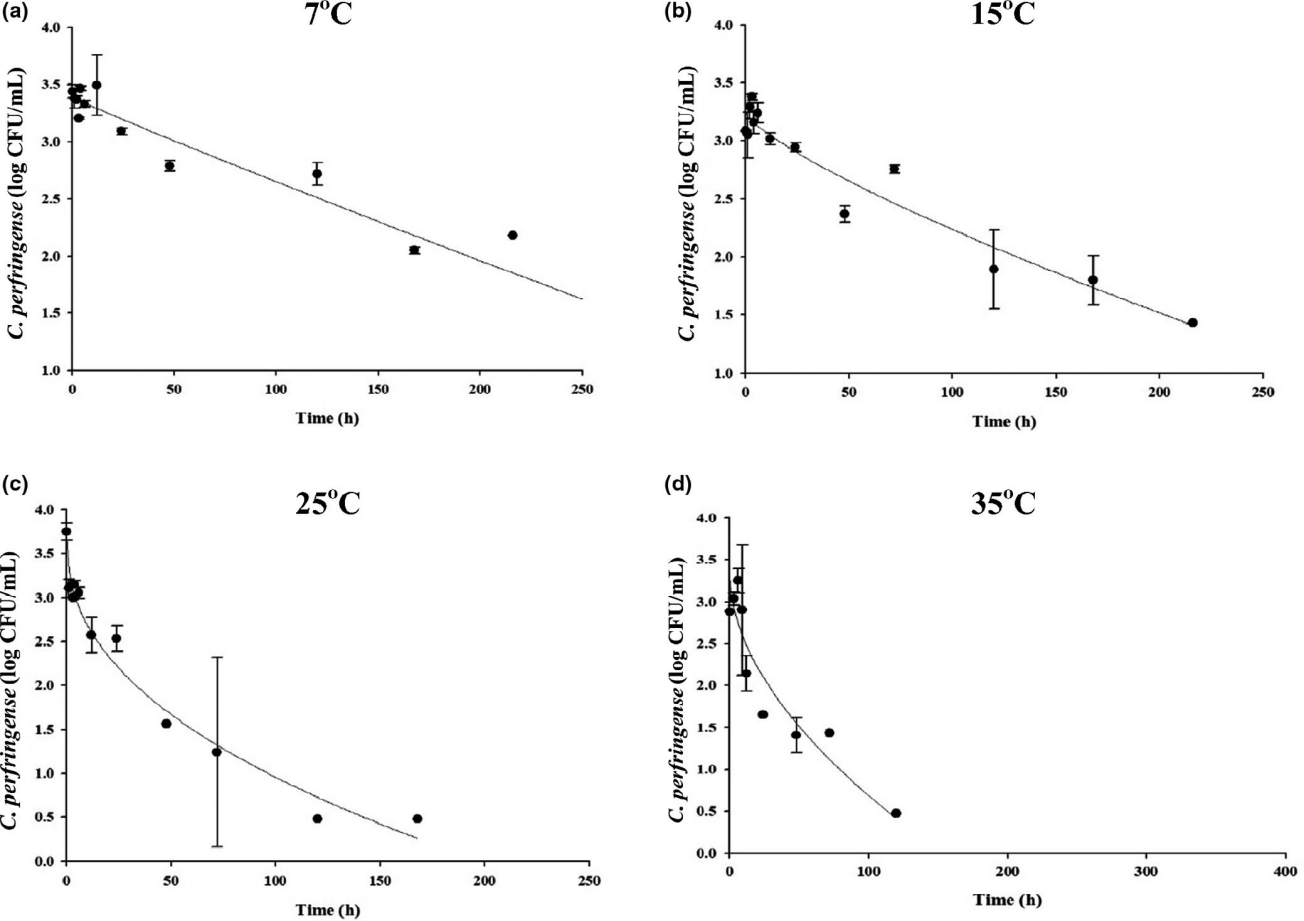
Fitted for *Clostridium perfringens* cell counts in soy sauce with primary models during storage at 7, 15, 25, and 35°C

To describe the *C. perfringens* cell count changes in soy sauce during transportation, storage, and display, the predictive models were developed. *C. perfringens* cell counts were collected at 7, 15, 25, and 35°C. The data were then fitted with the Weibull model to calculate δ and *ρ* values. *R^2^* values were 0.824–0.935, indicating that the developed model was appropriate to predict the cell counts as storage time increase from 7–35°C. The developed primary model showed that δ (the time needed to decrease 1 log CFU/ml) decreased as temperature increased (Figure 2, Table 1). This means that the increase in the storage temperature, the shorter the time it takes for 1 log CFU/ml reduction of *C. perfringens* cell counts in the soy sauce. Also, the *ρ* values ranged from 0.18 to 0.89 (Table 1). To evaluate the effect of temperature on δ and *ρ* values, a secondary model was developed, using the exponential growth model. The models were δ = 1/(−0.0057)+(0.0043** × **exp [0.1319** × **T]) and *ρ* = 1/(1.0196)+(0.0539** × **exp[0.1274** × **T]) with 0.942 and 0.985 of *R^2^*, respectively, indicating that the secondary models were developed appropriately to describe the changes of the parameters as a function of temperature. Validation of model performance showed that *RMSE* values were 0.403 and 0.854 at 20 and 30°C, respectively. *B_f_* and *A_f_* were 1.07 and 1.19 at 20°C, and 1.40 and 1.40 at 30°C. This indicates that the developed predictive models were appropriate to predict the *C. perfringens* cell counts in soy sauce during transportation and storage.

**TABLE 1:** Parameters from the Weibull model *Clostridium perfringens* fitted to cell counts in soy sauce during storage at 7, 15, 25, and 35°C

Parameters	Temperature (°C)			
	7	15	25	35
δ (hr)	91.91 ± 64.91	89.54 ± 14.04	8.89 ± 1.68	2.44 ± 0.65
*ρ*	0.89 ± 0.09	0.71 ± 0.10	0.43 ± 0.01	0.18 ± 0.02

### 3.3 Time and temperature for transportation, storage, and display

To predict changes in *C. perfringens* cell counts with the predictive model, during transportation, storage, and display time and temperature data are necessary. Hence, time and temperature for transportation and storage of soy sauce were collected. The probabilistic distribution for the temperature of the transport vehicle was appropriate with the Weibull distribution (RiskWeibull [1.3219, 2.8404, RiskShift (3.1093), RiskTruncate (2.2, 40)]) and with the Pert distribution with parameters (0.5, 3, 5) for the transport time. The probabilistic distribution showed that the mean temperature of the soy sauce was 5.7°C during transportation from production to sale. The products were then stored at 22–26°C for 0.1–48 hr. Thus, the temperatures were fitted to the Pert distribution (RiskPert [22, 25, 26]), and the storage time was fitted to the uniform distribution (RiskUniform [0.1, 48]). The probabilistic distribution for the display temperature was appropriate with loglogistic distribution (RiskLogLogistic [16.608, 5.6198, 14.584, RiskTruncate (2.2, 40)]), and the display time was fitted to the uniform distribution with parameters (0, 336).

### 3.4 Amounts and percentage of soy sauce consumption

**FIGURE 3:**
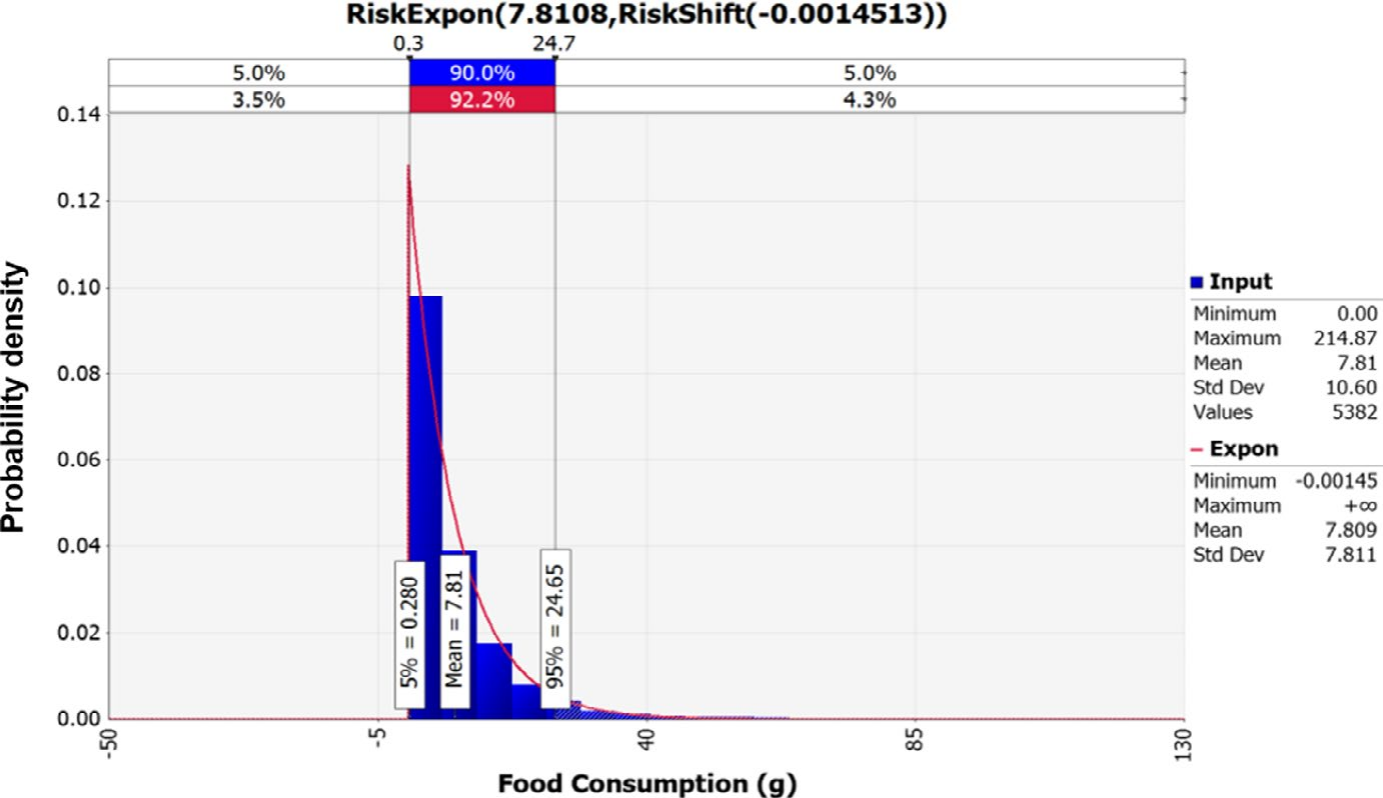
Probabilistic distribution for the intake of soy sauce in Korea

Raw data for daily consumption amounts of soy sauce extracted from KNHANES (Korea Centers for Disease Control & Prevention, 2017) were fitted with @RISK. Exponential distribution (RiskExpon [7.8108, RiskShift [−0.0014513], RiskTruncate (0, 215)]) was selected to be the appropriate probabilistic distribution for the consumption of soy sauce, and the probabilistic distribution showed that the average daily consumption of soy sauce was 7.81 ml (Figure 3) with 81.2% of consumption percentage in Korea.

### 3.5 Dose‐response model

For the dose‐response model for *C. perfringens*, there was only one model, which was the exponential model [*p* = 1−exp (−*r* **×** *N*), *r* = 1.82** × **10^−11^] developed by Golden et al. (2009), where P is the probability of illness, *r* is the probability of *C. perfringens* cells, causing foodborne illness, and *N* is the *C. perfringens* cell number (CFU/serving) ingested. Hence, this model was used in a simulation model.

### 3.6 Risk characterization

**FIGURE 4:**
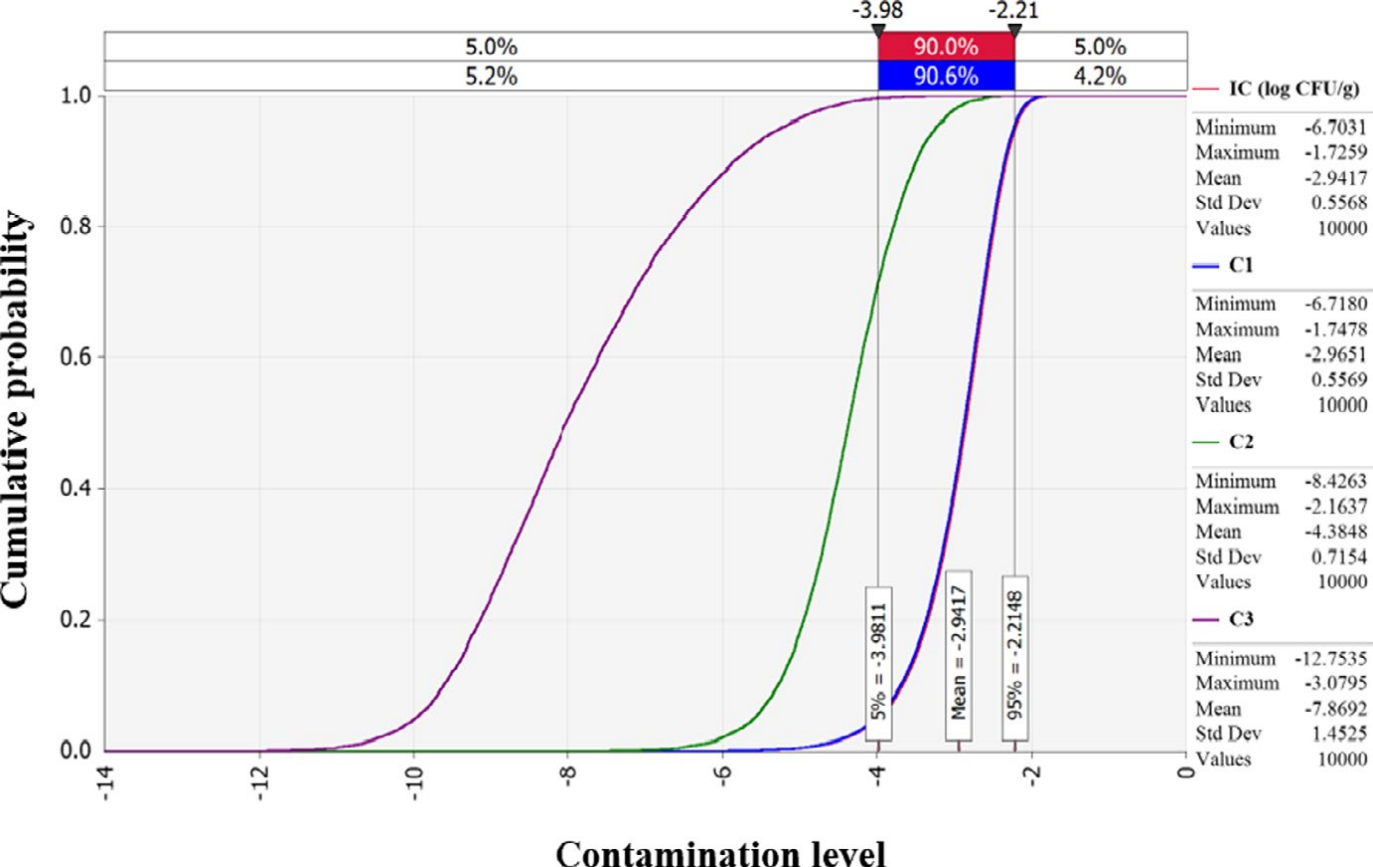
Changes in *Clostridium perfringens* contamination levels in soy sauce predicted by distributions during transportation

**FIGURE 5:**
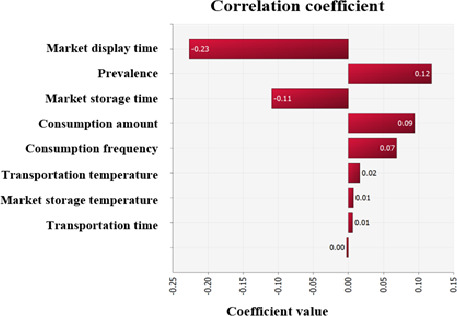
Correlation coefficients for risk factors affecting the probability of *Clostridium perfringens* illness per person per day by soy sauce consumption

Employing estimated *C. perfringens* contamination level, predictive models simulating *C. perfringens* cell counts with the probabilistic distributions of temperature and time, probabilistic distribution of consumption amounts, consumption percentage, and dose response model, the simulation model was prepared as shown in Table 2. The cumulative density determined by this simulation showed that estimated *C. perfringens* cell counts decreased gradually from initial contamination (IC) to display (C3) (Figure 4). The simulation result showed that the risk of *C. perfringens* per person per day through soy sauce consumption was 1.7** × **10^−16^ in Korea (Table 3). Also, the risk has a positive correlation with prevalence, consumption amount, and consumption percentage. On the other hand, it shows a negative correlation with market display time and market storage time. (Figure 5). In a study by Ko et al. (2012), the probabilities of *C. perfringens* foodborne illness per person per day for ham and sausage products was estimated as 3.97 × 10^−11^ ± 1.80 × 10^−9^. Also, the probability of *C. perfringens* foodborne illness by cheese consumption for natural and processed cheeses was 9.57 × 10^−14^ and 3.58 × 10^−14^ per person per day, respectively (Lee et al., 2016). Comparing these risks with the risk estimated in our study the risk of *C. perfringens* foodborne illness from soy sauce consumption was relatively low. This result might be caused by high salt concentration of soy sauce, which decrease *C. perfringens* cell counts are suggested in many studies (Chhetri & Karki, 2014; Gibson & Roberts, 1986; Juneja & Marmer, 1996).

**TABLE 2:** Simulation model for calculating the risk of *Clostridium perfringens* through soy sauce intake with @RISK

Input model	Unit	Variable	Formula	References
Product				
Pathogens contamination level				
*C. perfringens* prevalence		PR	=RiskBeta (1,491)	This research; Vose (1997)
Initial contamination level	CFU/ml	C	=‐LN (1 − PR)/1 ml	Sanaa et al. (2004)
	log CFU/ml	IC	=logC	
Market				
Transportation				
Transportation time	hr	Time_trans_	=RiskPert (0.5, 3, 5)	Personal communication^a^; This research
Food temperature during transportation	°C	Temp_trans_	=RiskWeibull (1.3219, 2.8404, RiskShift [3.1093], RiskTruncate [2.2, 40])	Personal communication; This research
Death				
Delta	hr	δ	=1/([−0.0057] + 0.0043 × exp [0.1319 × Temp_trans_])	This research
*ρ*		ρ	=1/(1.0196 + 0.0539 × exp (0.1274 × Temp_trans_))	This research
*C. perfringens* survival model	log CFU/ml	C1	=IC−(Time_trans_/δ) ^ρ^	Mafart et al. (2002)
Storage				
Storage time	hr	Time_Mark‐st_	=RiskUniform (0.1, 48)	Personal communication; This research
Food temperature during storage	°C	Temp_Mark‐st_	=RiskPert (22, 25, 26)	Personal communication; This research
Death				
Delta	hr	δ	=1/([−0.0057] + 0.0043 × exp [0.1319 × Temp_Mark‐st_])	This research
*ρ*		ρ	=1/(1.0196 + 0.0539 × exp [0.1274 × Temp_Mark‐st_])	This research
*C. perfringens* survival model	log CFU/ml	C2	=C1−(Time_Mark‐st_/δ)^ρ^	Mafart et al. (2002)
Display				
Display time	hr	Time_Mark‐dis_	=RiskUniform (0, 336)	Personal communication; This research
Food temperature during display	°C	Temp_Mark‐dis_	=RiskLogLogistic (16.608, 5.6198, 14.584, RiskTruncate [2.2, 40])	Personal communication; This research
Death				
Delta	hr	δ	=1/([−0.0057] + 0.0043 × exp [0.1319 × Temp_Mark‐dis_])	This research
*ρ*		ρ	=1/(1.0196 + 0.0539 × exp [0.1274 × Temp_Mark‐dis_])	This research
*C. perfringens* survival model	log CFU/ml	C3	= C2−(Time_Mark‐dis_/δ) ^ρ^	Mafart et al. (2002)
Consumption				
Daily consumption average amount	ml	Consump	=RiskExpon (7.8108, RiskShift (−0.0014513), RiskTruncate [0, 215])	KCDC. (2017)
Daily consumption percentage	%	ConFre	Fixed 81.2	KCDC. (2017)
		CF(0)	=1–81.2/100	KCDC. (2017)
		CF(1)	=81.2/100	KCDC. (2017)
		CF	=RiskDiscrete ({0, 1},{CF(0), CF(1)})	KCDC. (2017)
		Amount	=IF (CF = 0, 0, Consump)	KCDC. (2017)
Dose‐response				
*C. perfringens* amonut	CFU	*N*	=10^C3^ × Amount	Golden et al. (2009)
Parameter of r		r	=Fixed 1.82 × 10^–11^	
Risk				
Probability of illness/person/day		Risk	=1−exp (−r × *N*)	Golden et al. (2009)

**TABLE 3:** Probability of *Clostridium perfringens* foodborne illness per person per day with consumption of soy sauce

	5%	25%	50%	95%	99%	Mean
Probability of illness/person/day	0	0	0	0	3.9 × 10^–15^	1.7 × 10^–16^

## 4 CONCLUSIONS

In conclusion, the risk for *C. perfringens* foodborne illness soy sauce consumption in Korea is low, because although soy sauce is consumed widely, *C. perfringens* prevalence in soy sauce is low, and *C. perfringens* cell counts decreased during transportation, storage, and display.

## CONFLICT OF INTEREST

The authors declare that they have no conflict of interest.
